# The Role of Gly533 and Lys729 Residues in the Catalytic Activity and Friedelin Production of *Monteverdia ilicifolia* and *Populus davidiana* Synthases

**DOI:** 10.1021/acsomega.6c07069

**Published:** 2026-10-22

**Authors:** Weslei B. Botero, Vinícius Bonatto, Luis F. de Oliveira, Rafael V.C. Guido, Cleslei F. Zanelli, Sandro R. Valentini, Tatiana M. Souza-Moreira, Maysa Furlan

**Affiliations:** † Institute of Chemistry, 153998 São Paulo State University - UNESP, Araraquara, São Paulo 14800-901, Brazil; ‡ São Carlos Institute of Physics, 28133 University of São Paulo - USP, São Carlos, São Paulo 13566-590, Brazil; § School of Pharmaceutical Sciences, 28108 São Paulo State University - UNESP, Araraquara, São Paulo 14800-901, Brazil

## Abstract

Friedelin is a pentacyclic triterpene that exhibits a wide range of biological potential and is the precursor of bioactive quinonemethide triterpenes such as celastrol and pristimerin. This compound is synthesized by a specific oxidosqualene cyclase named friedelin synthase (FRS). This enzyme converts 2,3-oxidosqualene through rearrangement steps and promotes the production of a terpene with a ketone group at position C-3. Despite this, the precise mechanism of this oxidation remains unclear, highlighting the need to unveil FRS amino acid residues that are correlated with this specificity, catalytic efficiency, and friedelin production. For this reason, we performed site-directed mutagenesis in key residues and evaluated their production after heterologous expression in *S. cerevisiae*using GC-MS. Using multiple sequence alignment, we selected the residues Lys729, Tyr609, and Gly533 from *Monteverdia ilicifolia* FRS and correlated residues in *Populus davidiana* FRS for several mutations. Our results showed that the Lys729 residue is essential for enzyme activity and stability, while Tyr609 is not mandatory for friedelin production. Gly533Ala resulted in a notable improvement in the production of friedelin in *Mi*FRS (3.633 ± 0.203 mg/L) and *Pd*FRS (1.535 ± 0.169 mg/L) in comparison with wild type enzymes. Further molecular dynamics simulations showed that this particular mutation does not change significantly the structure of the enzyme but allows closer and stronger interactions that result in an increase in the amount of product formed by the mutant enzyme. In general, this work improves the understanding of residue interactions and shows a promising outlook for large-scale production of this compound.

## Graphical abstract

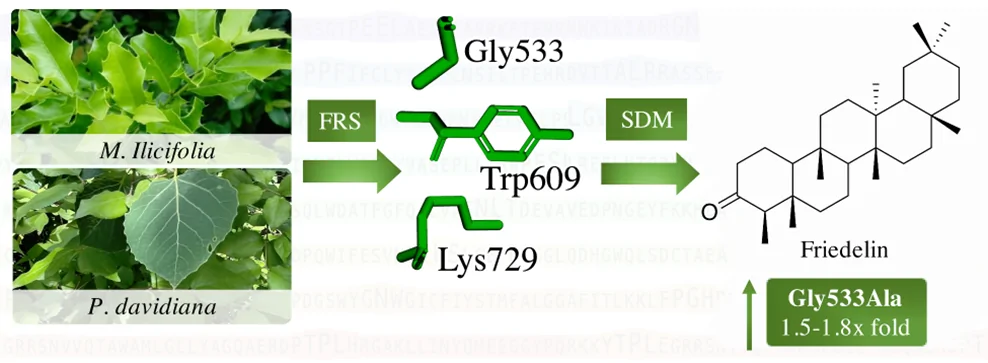

## 1 Introduction

Friedelin (friedelan-3-one) is a plant-derived pentacyclic triterpene characterized by a ketone group at the C-3 position. This compound is produced by a diverse range of plant species, such as *Celastraceae*, *Clusiaceae*, *Euphorbiaceae*, and *Salicaceae*.^1^ The structure of friedelin was first elucidated in 1956 from the bark of the cork oak, *Quercus suber*.^2^ However, it was not until 2010 that the enzyme responsible for its biosynthesis, friedelin synthase, was first cloned from *Kalanchoe daigremontiana*.^3^ Subsequently, research following the initial discovery has characterized friedelin synthase in a diverse range of species, leading to sequences deposited in the GenBank database from *Monteverdia ilicifolia*,^4^ *Populus davidiana*,^5^ *Tripterygium wilfordii*, and *Quercus suber*.^6,7^ Other putative friedelin synthases have been identified through sequence similarity in *Gymnosporia heterophylla*,^8^ *Pyrus bretschneideri*, and *Salix suchowensis*.^9,10^

Regarding its pharmacological potential, friedelin exhibits a wide range of activities, such as gastroprotective and anti-inflammatory,^11^ antimicrobial,^12,13^ anticancer,^14,15^ antioxidant, and hepatoprotective.^16,17^ In addition, this compound is the precursor of bioactive quinonemethide triterpenes (QMTs), such as celastrol from *T. wilfordii* and pristimerin from *M. ilicifolia*, known for their antiobesity, antiprotozoal, and anticancer activities.^18–21^

As with other triterpenes, friedelin biosynthesis initiates with the cyclization of 2,3-oxidosqualene, a reaction catalyzed by the specific oxidosqualene cyclase (OSC), friedelin synthase. This enzyme opens the epoxide ring to generate a carbocation intermediate that undergoes a series of rearrangements at different positions, with the final triterpene product depending on how the enzyme guides the cascade and stabilizes specific carbocation intermediates.^22,23^ While these transient cationic species have not been directly isolated, their occurrence is supported by product profiles of mutant enzymes and QM/MM calculations.^24,25^

Friedelin synthase (FRS) is the sole enzyme capable of driving the most extensive rearrangement cascade, resulting in the production of a ketone at the C-3 position. Recent studies suggest that the conversion from friedelin to celastrol is mediated by the C-3 carbonyl group of friedelin, a key feature introduced by the action of friedelin synthase, as shown in Figure 1.^24,26^

**Figure 1:**
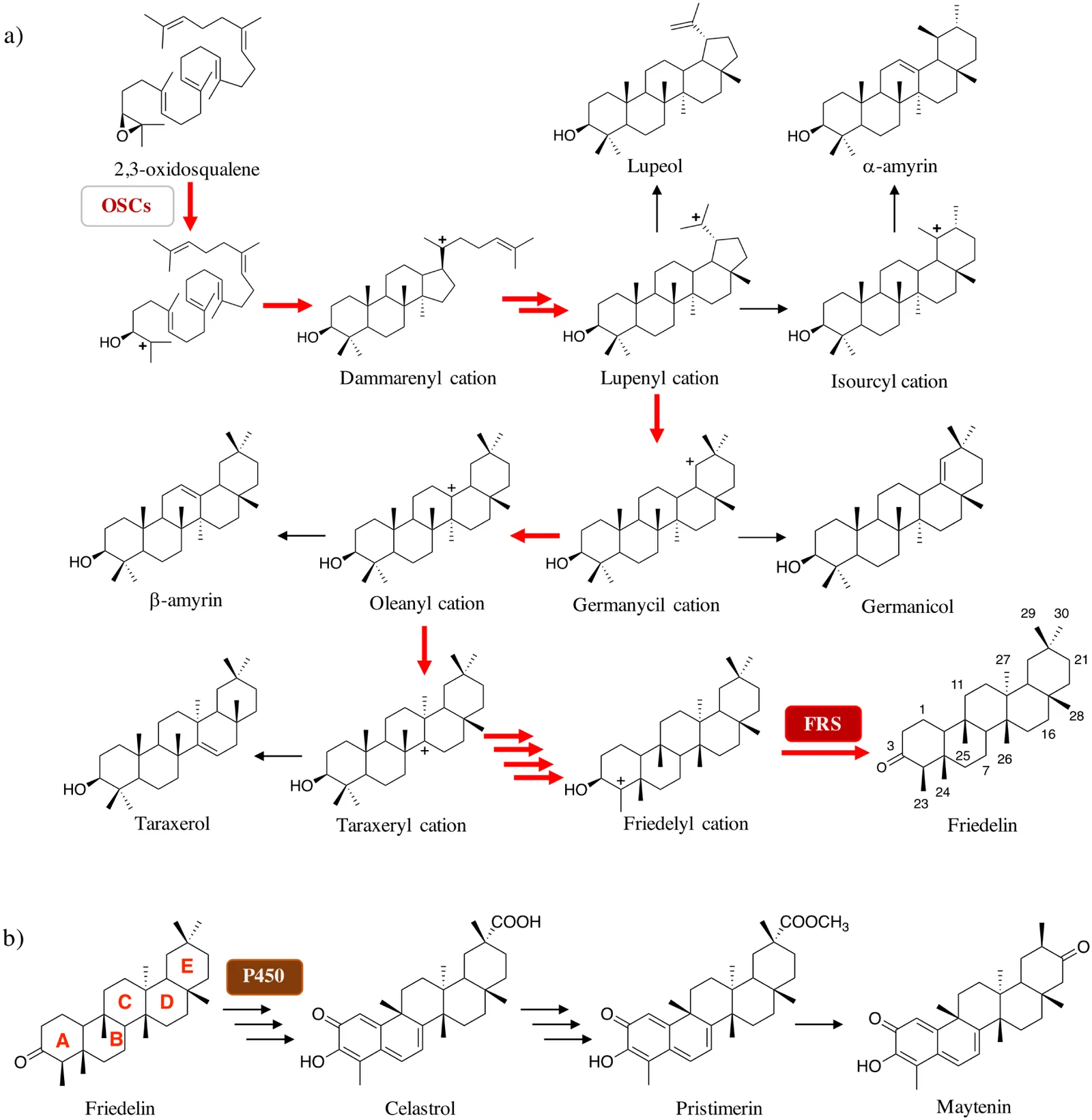
Proposed mechanism of cyclization of 2,3-oxidosqualene into a) friedelin and b) the production of quinonemethide triterpenes from it. Red arrows indicate the rearrangements leading to friedelin, while black arrows show triterpenes produced in earlier steps and quinonemethides production.

Despite this, the precise mechanism of this cyclization remains unclear, highlighting the need to elucidate friedelin synthase (FRS) specificity and devise strategies to increase production of the target compound.

The chemical synthesis of friedelin is complex and results in low yields.^27^ However, the enzyme sequence heterologously expressed in microorganisms such as *Saccharomyces cerevisiae* can easily promote compound production and can be optimized by metabolic engineering of the host or by improving the enzyme’s catalytic output.^24,28–30^ In this context, site-directed mutagenesis can be employed to investigate both enzyme structure–function relationships and production improvements.^31,32^

Several mutagenesis studies have been performed with OSCs over the years, leading to the identification of key residues for the catalytic efficiency and function. On the basis of homology modeling with human lanosterol synthase, approximately 98 residues have been mutated in more than 25 OSCs to date.^33,34^

A comprehensive review was recently published addressing the presence of motifs and conserved regions directly related to carbocation stabilization, cyclization, and product selectivity. Examples include the Asp-Cys-Thr-Ala-Glu (DCTAE), Met-(Trp/Leu)-Cys-(Tyr/His)-Cys-Arg (M­(W/L)­C­(Y/H)­CR), and Gln-Trp (QW) motifs, as well as changes in residues at the C-terminal region.^34^

However, up to now, we still need to understand which key residues led to the specific production of the ketone moiety in friedelin and which residues are directly related to its production. Therefore, the aim of this work was to evaluate the triterpene specificity and yield of friedelin synthase from *M. ilicifolia* and *P. davidiana* through site-directed mutagenesis, seeking to understand the friedelin structural specificity and quantify the production of wild and mutated sequences by *Saccharomyces cerevisiae* expression. Besides, we used molecular dynamics to suggest that the mutation Gly533Ala in the *M. ilicifolia* enzyme is responsible for the tight stabilization of aromatic residues in the active site, leading to a 1.8-fold increase in friedelin production.

## 2 Results and Discussion

### 2.1 Determination of Friedelin Synthase Mutants

To evaluate the residues involved in friedelin synthase (FRS) function and specificity, the FRS enzymes from *M. ilicifolia* (GenBank accession number: AVR49025.1; *Mi*FRS) and *P. davidiana*­(GenBank accession number: ART66198.1; *Pd*FRS) were selected to perform a multiple sequence alignment (MSA) of their primary sequences against other OSCs, including other friedelin-producing enzymes, pentacyclic triterpene-producing α- and β-amyrin synthases, germanicol, glutinol, lupeol, and taraxerol synthases, and tetracyclic triterpene cycloartenol synthases. To evaluate possible amino acids related to the C-3 oxidation, the enzymes shionone synthase from *Aster tataricus* (*At*SHS) and astertarone A synthase from *Brassica rapa* (*Br*ASS) were also used in the alignment input (GenBank accession numbers for all sequences are presented in Table S1, Supporting Information) and the clustering of the sequences is shown in the phylogenetic tree (Figure S1, Supporting Information). After evaluating the MSA, we could depict some residues in conserved regions that are different among the clustered enzymes. To illustrate, Figure 2 shows the logo of alignment around different residues in conserved regions as candidates for structure–function correlation in the enzymes.

**Figure 2:**
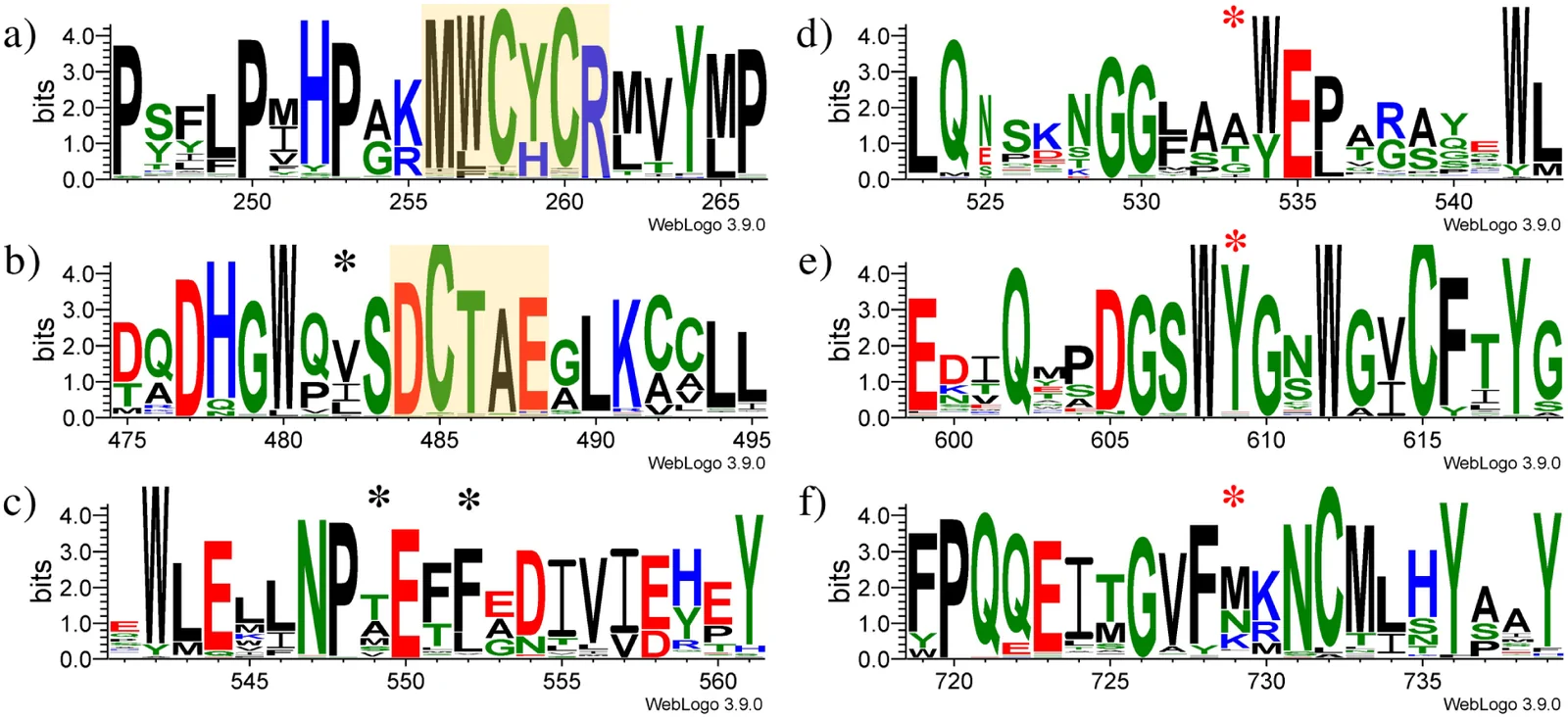
WebLogo overview of multiple sequence alignment (MSA) with a) conserved region M­(W/L)­C­(Y/H)­CR, b) conserved region DCTAE and 482 position, c) mutated positions 549 and 552, d) selected residue 533, e) selected residue 609, and f) selected residue 729. The conserved motifs over OSCs are highlighted in yellow; referenced mutations positions are indicated with a black asterisk, and new mutations are indicated with a red asterisk.

From the alignment, FRS sequences showed more than 66% identity. Notably, the FRS from *M. ilicifolia*showed the highest identity (above 93%) to the monofunctional *Tw*OSC4 of *T. wilfordii*, with identities above 87% to multifunctional FRS *Tw*OSC1­(2) and *Tw*OSC3­(2) FRS of the same species. Other FRSs, such as *Kd*FRS, *Qs*OSC3, and *Pd*FRS showed identities of about 65%, 67%, and 73%, respectively. *M. ilicifolia* and *T. wilfordii* are plants belonging to the same family, while *K. daigremontiana*, *Q. suber*, and *P. davidiana* each belong to a different family. In that sense, as already discussed, triterpene synthases have been shown to diverge in the same species from an ancestral gene, and their homology is greater among enzymes more closely correlated phylogenetically than across enzymes with the same activity from other species.^35^ Enzymes related to C-3 oxidation for shionone from *At*SHS and astertarone *Br*ASS production showed about 56–68% identity to FRS, while higher identities than 70% were observed among other pentacyclic triterpene cyclases.

FRS sequence identities drop down to 70% to 60% to α-amyrin, β-amyrin, lupeol, and taraxerol synthases, and then identity is around 50% to the tetracyclic cycloartenol synthases. Nonetheless, it is interesting to observe highly conserved segments across all synthases (Figures S2–S3, Supporting Information). Highly conserved motifs in pentacyclic triterpene synthases comprise the DCTAE and M (W/L)­C­(Y/H)­CR and four QW segments. A variety of studies have been carried out on the DCTAE motif, since this motif is in the active site of OSCs, being the aspartate residue responsible for the initial protonation in the rearrangement cascade shown in Figure 1.^6,36^ M­(W/L)­C­(Y/H)­CR is another motif vastly studied, characterizing its importance in facilitating D- and E-ring formation through cation−π interactions, and the presence of a Tyr or His residue for production of tetracyclic and pentacyclic residues.^37,38^

The importance of some other residues was investigated for the specificity and/or productivity of OSC enzymes. For example, it was observed that a leucine close to the DCTAE motif (Leu482 in *Mi*FRS and Leu486 in *Tw*OSC1) plays a key role in product selectivity and catalytic efficiency by providing steric accommodation for the oleanyl intermediate, thereby enabling the extensive rearrangement required to produce friedelin.^39^ This leucine is only present in friedelin synthases and shionone synthase, while a valine residue is present in astertarone and other pentacyclic triterpenes and an isoleucine is in cycloartenol synthases (Figure 2, item b). Interestingly, site-directed mutations Leu482Val and Leu486Val led to high β-amyrin production besides friedelin, while the Leu482Thr mutation abolished friedelin production and only β-amyrin was detected by yeast heterologous production.^25,39^ *In silico* homology modeling, changing to a threonine led to recruitment of a water molecule to the active site, which interacted with the catalytic aspartate residue, thereby displacing and altering the stabilization of the oleanyl cation, as well as blocking the rearrangements of the methyl group from A-ring.^39^

Our group has also investigated the function of two residues exclusive in *Mi*FRS and *Pd*FRS, Met549 and Leu552. By changing Met549 to a serine residue (present in human OSC) and Leu552 to a phenylalanine common to other pentacyclic triterpene synthases (PTSs), α- and β-amyrin were also produced. Interestingly, friedelin production in Met549Ser was two times higher than the wild-type sequence, even with other PTS parallel production.^40^

Figure 2 (items d–f) also shows that the residues Gly533 and Lys729 in *Mi*FRS and Gly530 and Lys726 in *Pd*FRS are mostly conserved in 3-keto OSCs in comparison with other enzymes, which indicates possible sites for better understanding friedelin synthesis. Therefore, after preparing the mutants, triterpene production was evaluated by gas chromatography coupled to mass spectrometry (GC-MS), with the chromatograms presented in Figures S4–S5­(Supporting Information). Molecular dynamics (MD) simulations were calculated to reach a more reliable analysis of the enzyme mutation Gly533Ala (*Mi*FRS^Gly533Ala^) improvement in the level of friedelin production.

### 2.2 *Mi*FRS^Lys729^ and *Pd*FRS^Lys726^ are Essential for Enzyme Activity

Lysine residues (*Mi*FRS^Lys729^ and *Pd*FRS^Lys726^) were mutated due to its conservation among friedelin synthases, including glutinol synthase *Kd*GLS, which, besides glutinol, produces friedelin (as well β-amyrin and taraxerol). We observed that the presence of this residue was important to the whole enzyme activity, since all the mutants led to the loss of activity and did not present triterpene production (Table 1). Evaluated mutants were obtained by changing the lysine to methionine, arginine, alanine, glutamic acid, and tryptophan.

**Table 1:** Site-Directed Mutations, Observed Production, and Structural Modification

Enzyme	Product	Product Titer^a^ (mg·L^–1^)	Structural modification
*Mi*FRS	friedelin	2.024	wild type
*Pd*FRS	friedelin	1.024	wild type
*Mi*FRS^Lys729^ mutations	none	-	Lys residue is essential to enzyme activity
*Pd*FRS^Lys726^ mutations	none	-	
*Mi*FRS^Tyr609Ala^	friedelin	0.677	Aliphatic residue with a smaller steric bulk
*Pd*FRS^Tyr606Ala^	friedelin	1.205	
*Mi*FRS^Gly533Ala^	friedelin	3.633	Ala residue donates electronic density
*Pd*FRS^Gly533Ala^	friedelin	1.535	

a Titer is presented as milligrams of triterpene per liter of total inoculum. It was determined by GC-MS from whole-cell extracts of engineered *S. cerevisiae* cultured under standardized conditions across biological triplicates.

On the other hand, when Luo et al. (2023) evaluated the correspondent *Tw*OSC1^Lys732^ mutants, they observed almost the loss of friedelin production by the mutation Lys732Arg and an inversion and improvement in β-amyrin production with the Lys732Met mutant.^25^ Molecular mechanics and molecular dynamics simulations led to the analysis of one water molecule entering the active site and changing Tyr262 orientation in order to stabilize the oleanyl carbocation, producing β-amyrin, what was consistent with the effect observed with *Mi*FRS Leu482Thr mutant and a water displacement of residues in the active site. These opposite results are in accordance with mixed results observed when mutating the C-terminus of different triterpene synthases.^34^ Some mutations led to specificity loss while others abolished enzyme activity, indicating that the C-terminus is important to the enzyme activity but could not exhibit a conserved function across enzymes.

### 2.3 *Mi*FRS^Tyr609^ and *Pd*FRS^Tyr606^ Are Conserved but Not Mandatory for Friedelin Production

Tyrosine, corresponding to position 609 in *Mi*FRS and position 606 in *Pd*FRS, is inside a highly conserved segment among OSCs and is also conserved (Figure 2, item e). It localizes close to Trp608, which corresponding Trp612 in *Et*AS was mutated to Phe and Tyr, leading to germanicol and lupeol formation, respectively; in the corresponding Trp616 of *Pg*DS the change to alanine diminished dammarenediol II production.^41,42^ Change of Trp612 to Phe in *Ia*AS1 abolished α-amyrin production while *Ia*AS2 produced more β-amyrin than α-amyrin.^43^ Tyr606 in *Tw*OSC1 was calculated as a potential candidate to increase triterpene yield by changing to a phenylalanine residue, but after the mutation the triterpenes production dramatically dropped.^25^ When we changed Tyr609 in *Mi*FRS and Tyr606 in *Pd*FRS to alanine, a very small aliphatic amino acid, friedelin production was still active and did not change significantly in *Pd*FRS but decreased almost 3-fold in *Mi*FRS (from 2.024 ± 0.169 mg·L^–1^ to 0.677 ± 0.072 mg·L^–1^). These results indicate the contribution of the tyrosine residue in the proper cation shifting, since even changing to another aromatic amino acid, such as phenylalanine, decreased triterpene yield was observed.^25^

### 2.4 Alanine Residues in Mutations *Mi*FRS^Gly533Ala^ and *Pd*FRS^Gly530Ala^ Improved Friedelin Production

Glycine at position 533 in *Mi*FRS and 530 in *Pd*FRS is present in friedelin synthase enzymes and in the multifunctional friedelin/glutinol synthase *Kd*GLS (Figure 2, item d). The other two 3-keto synthases, the shionone and astertarone synthases, have isoleucine and alanine at the corresponding position, while other PTSs exhibit alanine at the same position.

Therefore, Gly533 and Gly530 were changed to Ala in expectation of another triterpene production. Surprisingly, this resulted in a statistical significative improvement of 1.8- and 1.5-fold in friedelin production in *Mi*FRS (3.633 ± 0.203 mg/L) and *Pd*FRS (1.535 ± 0.169 mg/L) (Table 1). The same result was observed by Luo et al. (2023), where they simulated quantum mechanics/molecular mechanics (QM/MM) to probe the cation cascade rearrangement until friedelin in *Tw*OSC.^25^ Their analysis suggested that the corresponding site *Tw*OSC^Gly536^ is in an outer loop of the enzyme and could contribute to the rigidity of the site but not to the rearrangement mechanism.

As described before, mutant *Mi*FRS^Met549Ser^ increased friedelin production 2-fold and led to the detectable production of β- and α-amyrin, while *Tw*OSC1^Tyr502Glu^ mutant increased friedelin production by 1.4-fold but maintained similar yields of β-amyrin and α-amyrin.^6,40^ These results, along with those described in this paper, are promising starting points to enhance and scale up the yield of friedelin in *S. cerevisiae*.

In this way, the combination of heterologous production and the use of computer molecular dynamics simulations of the enzyme helped to understand the residue interactions and showed a promising outlook for large-scale production of this compound.

### 2.5 Molecular Dynamics Simulations Reveal Differences in Gly533Ala Active-Site Conformational Sampling

The molecular dynamics (MD) simulations revealed consistent differences between the wild type (WT) and Gly533Ala mutant. The global stability of the complexes was evaluated by root-mean-square deviation (RMSD; complex and ligand) and root-mean-square fluctuation (RMSF) over all replicates (Figures S6–S10 and Supporting Information), and they showed no clear systematic differences between the WT and Gly533Ala systems. A representative structure of each system is shown in Figure 3.

**Figure 3:**
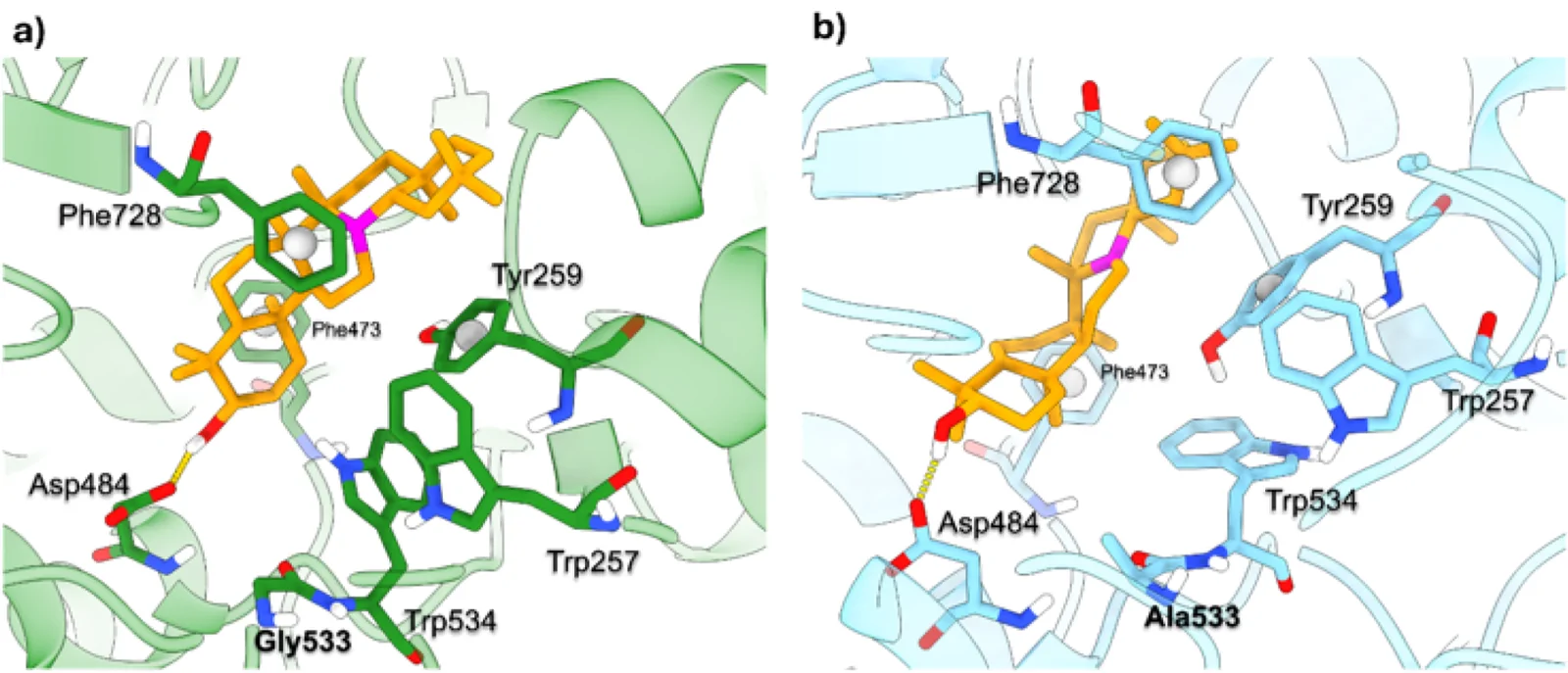
Binding mode of the oleanlyl cation in the active site of (a) the wild type enzyme (green) and (b) the Gly533Ala mutant (blue). The substrate is shown in orange, with the positively charged carbon of the carbocation highlighted in magenta. Key residues are represented in stick format, and the centroids of the aromatic residues are depicted as light gray spheres. The hydrogen-bond interaction with Asp484 is indicated by yellow dashed lines.

A key observation concerns the distance between the alcohol group of the ligand and the catalytic residue Asp484. In the WT system, the mean distances to the OD1 and OD2 atoms of Asp484 were 4.2 Å and 4.5 Å, respectively. In contrast, the Gly533Ala mutant presented shorter mean distances of 3.5 Å and 3.4 Å, indicating that the selected oleanyl cation pose sampled shorter distances from Asp484 in the Gly533Ala system. These distances do not establish greater energetic stabilization or an effect on the reaction barrier.

Another important difference arises in the positioning of the ligand relative to the aromatic residues Phe728 and Tyr259. In the WT system, the average distances between the carbocation center (magenta in Figure 3) and these residues were 4.7 Å (Phe728) and 5.0 Å (Tyr259). In the Gly533Ala system, these values decreased to 3.50 Å and 3.4 Å, respectively. The shorter distances are geometrically consistent with possible cation−π interactions involving Tyr259 and Phe728. However, their energetic contributions cannot be determined from distance measurements alone.^44,45^ This type of interaction could provide additional stabilization for the carbocation formed during the rearrangement step, thus favoring product formation.^46–50^

Moreover, the Gly533Ala substitution introduces a bulkier side chain that may increase steric hindrance in the vicinity of Trp534 and Trp257 and may result in decreased flexibility of these residues. This subtle rearrangement of local packing could allow these tryptophan residues to adopt conformations that further support stabilization of the carbocation intermediates through aromatic cation−π interactions. Together, these effects provide a structural rationale for the experimental observation that the Gly533Ala variant produces more product than the WT enzyme. It is important to note that the simulations employed a classical fixed-topology force field and therefore did not model carbocation formation, bond rearrangement, or reaction barriers. The results represent conformational sampling conditional on the selected literature-guided oleanyl cation pose.^25,39^ They do not establish that this intermediate equilibrates during catalysis, identify a unique reactive pose, or exclude nonequilibrium progression along the carbocation cascade.

The pocket analysis with KVFinder provided consistent results across the 10 representative clusters extracted from the three replicates of each system. For the Gly533Ala mutant, the pocket presented a mean volume of 800 ± 89 Å^3^ and a mean surface area of 561 ± 49 Å^2^, while the WT showed a very similar mean volume of 790.06 Å^3^ (SD = 117.16) and a mean surface area of 560 ± 69 Å^2^. Likewise, the average hydropathy values were comparable between the systems (Gly533Ala: −0.70; WT: −0.72). The only noticeable difference was related to pocket depth, where the Gly533Ala system displayed a higher maximum (13 Å vs 9 Å) and average depth (7 Å vs 5 Å). These results indicate that the mutation does not drastically alter the overall shape, size, or polarity of the active site pocket. However, the slight increase in pocket depth in the Gly533Ala mutant may reflect subtle steric constraints introduced by the Ala side chain. This could indirectly affect the orientation of nearby aromatic residues, particularly Trp534 and Trp257, which, through cation−π interactions, may contribute to stabilizing carbocation intermediates during the reaction. The replacement of Gly by Ala at position 533 may thus introduce additional steric hindrance that favors closer positioning of these residues, thereby potentially enhancing their stabilizing role in the subsequent steps of the reaction.

## 3 Conclusions

In this study, the roles of three amino acid residues in friedelin synthase from *M. ilicifolia* and *P. davidiana* were evaluated through site-directed mutagenesis and heterologous expression in *S. cerevisiae*. Our findings showed that the lysine 729 residue in *M. ilicifolia*, located in the C-terminal region for 3-keto-OSCs, is essential for enzyme activity, and mutations led to no triterpene production.

A tyrosine in position 609 in *M. ilicifolia*friedelin synthase is a highly conserved residue but is not mandatory for friedelin production, with previous studies suggesting its role in stabilizing upstream cationic intermediates along the rearrangement cascade. Finally, a glycine residue in position 533 from *M. ilicifolia* FRS was also mutated, resulting in an unexpected increase of 1.5- to 1.8-fold production of friedelin when substituted for alanine. Molecular dynamics (MD) studies were also performed about this mutation and indicate the presence of shorter sampled distances between the modeled intermediate and several active-site residues, providing a structural hypothesis consistent with enhanced friedelin production.

In summary, this study allowed us to identify key residues for the activity and product yield of the friedelin synthase enzyme, resulting in promising data that may be used to further scale its production.

## 4 Materials and Methods

### 4.1 Cloning the Wild-Type Enzymes *Mi*FRS and *Pd*FRS

The coding sequences encoding friedelin synthases from *Monteverdia ilicifolia* (GenBank accession number: AVR49025.1) and *Populus davidiana* (GenBank accession number: ART66198.1) were commercially synthesized (GenOne Biotechnologies, Brazil) with codon optimization for expression in *S. cerevisiae*. Full-length coding regions were PCR-amplified with *Pfx* DNA Polymerase enzyme (Invitrogen, United States) using *att*B primers listed in Tables S2–S3 (Supporting Information). The amplified PCR products were resolved on 1% (w/v) agarose gels, purified using GeneJet Gel Extraction kit (Thermo Fisher Scientific, United States), and initially recombined into the pDONR 207 entry vector via Gateway BP Clonase II enzyme mix (Invitrogen, United States). Following sequence confirmation, the target genes were transferred into the yeast destination expression vector pESC-URA-DEST [P_*GAL1*_-*t*HMG1, P_*GAL10*_, *URA3*, 2 μ] using Gateway LR Clonase II enzyme mix (Invitrogen, United States) according to the manufacturer’s protocol.^51^

### 4.2 Site-Directed Mutagenesis Reactions

Site-directed mutagenesis was performed using 200 ng of plasmid DNA templates, Phusion High-Fidelity DNA Polymerase (New England Biolabs, United States), and mutagenic primers according to the single-primer amplification method.^52^ Following PCR amplification, parental methylated DNA templates were digested with the restriction enzyme *DpnI* (New England Biolabs, United States) at 37 °C for 1 h. The mutated DNA coding sequences in the recombinant plasmids were transformed into chemically competent *E. coli* DH5α cells. The success of each mutation in the DNA sequence of the enzyme was confirmed by Sanger sequencing using the primers listed in Tables S2–S3 (Supporting Information). Briefly, plasmids containing the mutated DNA were purified from transformed cells with the GeneJET Plasmid Miniprep Kit (Thermo Scientific, United States), sequenced using a Genetic Analyzer 3130 with the Big Dye Terminator v3.1 Cycle Sequencing Kit, and purified with the BigDye XTerminator Purification Kit (Applied Biosystems, United States). The mutation in DNA sequences obtained were identified by nucleotide alignment among the wild type and mutated sequences BLASTn tool (http://blast.ncbi.nlm.nih.gov/Blast.cgi).

### 4.3 Heterologous Expression in *S. cerevisiae*

Recombinant destination plasmids containing wild type or mutated friedelin synthase genes were transformed into the modified *Saccharomyces cerevisiae* strain CEN.PK113–5D (*MATa*, *MAL2–8c SUC2 ura3–52* P_*ERG7*_::P_*KEX2*_ *-ERG7*) using the standard lithium acetate/polyethylene glycol method.^53^ Transformants were selected and maintained in synthetic complete medium without uracil (SC-U) supplemented with 2% (w/v) glucose. For protein expression and triterpene production, single colonies were precultured overnight at 30 °C and 200 rpm in 5 mL liquid SC-U medium containing 2% (w/v) glucose. The precultures are inoculated in 10 mL of SC-U induction medium containing 2% (w/v) galactose to an initial optical density at 600 nm (OD_600_) of 0.1. Heterologous expression was carried out in biological triplicate at 30 °C and 200 rpm for 36 h.

### 4.4 Extraction and Quantitative Analysis from the Heterologous System

After induction, yeast cells were collected by centrifugation (4,000*g*, 10 min) and lyophilized to dryness. Total triterpenes were extracted from the cell pellets using an ultrasonic bath (USC-2800A, Unique, Brazil) with 7 mL CHCl_3_:MeOH (2:1, v/v) for 10 min. Phase separation was subsequently provided with the addition of 1.7 mL of 0.73% (w/v) aqueous NaCl solution. The lower organic phase containing triterpenes was collected, evaporated to dryness, and resuspended in 300 μL of chromatographic-grade CHCl_3_. A commercial standard of cholesterol (Sigma-Aldrich, United States) was added as an internal standard at a final concentration of 240 μg mL^–1^.

Chromatographic analysis was performed on a gas chromatography system coupled to a mass spectrometer (GC-MS, QP2020C W/O RP230 V, Shimadzu, Japan) using an HP-5 capillary column (30 m × 0.25 mm × 0.25 μm; Agilent Technologies, United States). The operational conditions were set as follows: injector temperature, 270 °C; heating ramp, from 200 to 290 °C (10 °C/min); trap temperature, 200 °C for 3 min; interface temperature, 290 °C for 18 min; injection volume, 1 μL; split-type injection mode, 1:10; carrier gas flow, 1.0 mL min^–1^; total analysis time, 30 min; and electron ionization mass spectra acquired in full-scan mode over an *m*/*z* range of 35–600.

Triterpene quantification was determined using an external calibration curve prepared with an authentic friedelin standard (Sigma-Aldrich, United States) across a concentration range from 0.97 to 500 μg mL^–1^. Product concentrations were normalized to the initial cultivation volume, reporting the volumetric friedelin output as a titer in mg L^–1^ of culture broth under standardized 36-h induction across independent biological triplicates.

### 4.5 Multiple Sequence Alignment and Logo

For phylogenetic and alignment analysis, a total of 77 amino acid sequences of triterpene synthases, including 3-keto OSCs, β-amyrin synthase (BAS), lupeol synthase (LUS), cycloartenol synthase (CAS), and other functional OSCs were used (Table S1, Supporting Information). Sequences were aligned using Clustal Omega (EMBL, England), and evolutionary distances were computed through MEGA11 using Neighbor-Joining method with default parameters, while bootstrap = 1000.^54,55^ Alignment images were created by using BoxShade (ExPasy, Switzerland) and the WebLogo 3 web tools.^56–58^

### 4.6 Molecular Dynamics Simulations

All the following steps were performed for both the wild type (WT) and the Gly533Ala mutant of *Mi*FRS. The initial models of both proteins were generated using AlphaFold2,^59^ based on the oxidosqualene cyclase (OSC) friedelin synthase from *Tripterygium wilfordii* Hook. f. (*Tw*OSC, PDB code: 8J5Z).^25,60^ Specifically, five AlphaFold2-PTM models were generated and evaluated using pLDDT, pTM, and PAE. The models showed mean pLDDT values of 94.8–95.4 and pTM scores of 0.929–0.937. The highest-ranked model, with a mean pLDDT of 95.37 and pTM of 0.937, was selected for system preparation. Residue-level pLDDT values were 95.81 for Trp257, 95.50 for Tyr259, 98.19 for Asp484, 97.62 for Gly533, 97.25 for Trp534, 88.94 for Phe728, and 89.06 for Lys729. Protonation states of amino acid residues were assigned with PDB2PQR,^60^ assuming a pH of 7.2. As a representative substrate, the oleanyl cation was chosen for modeling. Its 3D structure was generated from a SMILES string using OMEGA2. The ligand was docked into the active site of each protein using GOLD,^61^ defined by a 15 Å sphere centered on the α-carbon of Asp484. Docking poses were visually inspected and compared with previously reported results, and the most appropriate binding mode was selected for MD simulations. For MD simulations, ligand charges were derived using the RESP protocol with the Psi4 package.^62^ Protein parameters were taken from the ff14SB force field, and the ligand was parametrized using GAFF2. Each protein–ligand complex was solvated in a truncated octahedron box of TIP3P water molecules, with an appropriate number of Na^+^ counterions added to neutralize the system. The solvated WT system comprised 106,770 atoms, whereas the Gly533Ala system contained 113,472 atoms. Energy minimization was performed in stages: first restraining the protein and minimizing only hydrogen atoms, then water molecules and ions, followed by minimization of the entire system. Next, the systems were gradually heated from 0 to 300 K under the NVT ensemble using a Langevin thermostat, followed by NPT equilibration at 300 K. Finally, production runs of 300 ns were carried out in triplicate for each system, totaling 900 ns per protein (WT and Gly533Ala). All simulations were performed using AMBER24. Trajectory analysis was carried out with cpptraj and MDAnalysis.^63,64^ For structural clustering, MDANCE was used to generate 10 representative clusters with the k-Means++ approach over all replicates.^65^ To further characterize the binding sites, the KVFinder Python package was employed to calculate pocket volumes for each representative cluster.^66,67^

## Supplementary Material



## Notes

Supporting information (multiple sequence alignment input, phylogenetic three, primer sequences, and computational data) is available free of charge. Identification and GenBank accession numbers of all protein sequences selected for the multiple alignment (Table S1); primers used in sequencing and mutation of *Monteverdia ilicifolia* friedelin synthase (Table S2); primers used in sequencing and mutation of *Populus davidiana* friedelin synthase (Table S3); multiple sequence alignment of OSCs with highlighted residues used in site-directed mutagenesis (Figure S1); logo alignment of OSCs (Figure S2); phylogenetic tree of OSCs (Figure S3); chromatographic profile of *Mi*FRS wild-type enzymes and mutants produced in *S. cerevisiae* using GC-MS (Figure S4); chromatographic profile of *Pd*FRS wild-type enzymes and mutants produced in *S. cerevisiae* using GC-MS (Figure S5); root-mean-square fluctuations: (a) WT system and (b) Gly533Ala mutant (Figure S6); root-mean-square deviation along the MD simulations of the WT system (Figure S7); root-mean-square deviation along the MD simulations of the Gly533Ala system (Figure S8); distance between the hydroxyl group of the substrate and the OD1 atom of Asp484 during the MD simulations (Figure S9); and distance between the hydroxyl group of the substrate and the OD2 atom of Asp484 during the MD simulations (Figure S10) (PDF)

## Abbreviations

AMBER: assisted model building with energy refinement
CHCl_3_: methyl chloride
DNA: DNA
FRS: friedelin synthase
GAFF: general amber force field
GC-MS: gas chromatography coupled with mass spectrometry
GOLD: Genetic Optimization for Ligand Docking
K: kelvin
*m*/*z*: mass-to-charge ratio of an ion
MD: molecular dynamics
MDANCE: Molecular Dynamics Analysis with N-ary Clustering Ensembles
MeOH: methanol
MSA: multiple sequence analysis
NaCl: sodium chloride
NPT: Isothermal–Isobaric ensemble
NVT: canonical ensemble
OD: delta oxygen
OSC: oxidosqualene cyclase
PCR: polymerase chain reaction
PTS: pentacyclic triterpene synthase
QMT: quinonemethide triterpene
RESP: Restrained Electrostatic Potential
RMSD: root-mean-square deviation
RMSF: root-mean-square fluctuation
SC-U: synthetic complete minus uracil
SD: standard deviation
TIP3P: transferable intermolecular potential with 3 points
WT: wild type.
