# Supplementary material for: The Role of Gly533 and Lys729 Residues in the Catalytic Activity and Friedelin Production of Monteverdia ilicifolia and Populus davidiana Synthases
Source: ACS Omega. 2026 Oct 22;11(43):66970–9. doi: 10.1021/acsomega.6c07069 (PMC13737970; doi:10.1021/acsomega.6c07069)

Supplementary Information

The Role of Gly533 and Lys729 residues in the Catalytic Activity and Friedelin Production of *Monteverdia ilicifolia* and *Populus davidiana* Synthases

Weslei B. Botero [1], Vinícius Bonatto [2], Luis F. de Oliveira [2], Rafael V. C. Guido [2], Cleslei F. Zanelli [3], Sandro R. Valentini [3], Tatiana M. Souza-Moreira [3], Maysa Furlan [1].*

1 Institute of Chemistry, São Paulo State University - UNESP, 14800-901, Araraquara, São Paulo, Brazil.

2 São Carlos Institute of Physics, University of São Paulo - USP, 13566-590, São Carlos, São Paulo, Brazil.

3 School of Pharmaceutical Sciences, São Paulo State University - UNESP, 14800-901, Araraquara, São Paulo, Brazil.

Table S1. Identification and GenBank accession number of all protein sequences selected for the multiple alignment.

OSC	GenBank ID	Species	Product
MiFRS	AVR49025.1	*Monteverdia ilicifolia*	friedelin
MiFRS1	APG38073.1	*Monteverdia ilicifolia*	friedelin
MiFRS2	AVR49026.1	*Monteverdia ilicifolia*	friedelin
MiFRS3	AVR49027.1	*Monteverdia ilicifolia*	friedelin
MiFRS4	AVR49028.1	*Monteverdia ilicifolia*	friedelin
KdFRS	ADK35125.1	*Kalanchoe daigremontiana*	friedelin
PdFRS	ART66198.1	*Populus davidiana*	friedelin
TwOSC4	1HT64457.1	*Tripterygium wilfordii*	friedelin
MiCAS	APG38074.1	*Monteverdia ilicifolia*	cycloartenol
NtCAS1	AIY33888.1	*Nicotiana tabacum*	cycloartenol
WsOSC	ADG60271.1	*Withania somnifera*	cycloartenol
WsCAS	AOX15290.1	*Withania somnifera*	cycloartenol
AsCS1	CAC84559.1	*Avena strigosa*	cycloartenol
BpBPX1	BAB83085.1	*Betula platyphylla*	cycloartenol
BpBPX2	BAB83086.1	*Betula platyphylla*	cycloartenol
GgCAS1	BAA76902.1	*Glycyrrhiza glabra*	cycloartenol
KdCAS	ADK35127.1	*Kalanchoe daigremontiana*	cycloartenol
KcCAS	BAF73930.1	*Kandelia candel*	cycloartenol
LjOSC5	BAE53431.1	*Lotus japonicus*	cycloartenol
LcCAS1	BAA85266.1	*Luffa cylindrica*	cycloartenol
OsOSC2	BAH00370.1	*Oryza sativa*	cycloartenol
PgPNX	BAA33460.1	*Panax ginseng*	cycloartenol
PsPSX	BAA23533.1	*Pisum sativum*	cycloartenol

RsCAS	BAF73929.1	*Rhizophora stylosa*	cycloartenol
RcCAS	ABB76767.1	*Ricinus communis*	cycloartenol
ElCAS1	QBQ04199.1	*Euphorbia lathyris*	cycloartenol
TwOSC4(2)	AWK97869.1	*Tripterygium wilfordii*	cycloartenol
TwOSC6	AWK97868.1	*Tripterygium wilfordii*	cycloartenol
TwOSC1	QHT64454.1	*Tripterygium wilfordii*	β-amyrin
TcOSC3	QBO24613.1	*Taraxacum coreanum*	β-amyrin
TwOSC2	AWK97811.1	*Tripterygium wilfordii*	β-amyrin
GmBAS3	AHI17180.1	*Glycine max*	β-amyrin
ObAS1	AHJ10507.1	*Sweet Basil*	β-amyrin
WsOSC/BS	AGA17940.1	*Withania somnifera*	β-amyrin
AtLUP4	NP_178016.2	*Arabidopsis thaliana*	β-amyrin
AaBAS	ACA13386.1	*Artemisia annua*	β-amyrin
BpBPY	BAB83088.1	*Betula platyphylla*	β-amyrin
BgBAS	BAF80443.1	*Bruguiera gymnorrhiza*	β-amyrin
EtBAS	BAE43642.1	*Euphorbia tirucalli*	β-amyrin
GgBAS1	BAA89815.1	*Glycyrrhiza glabra*	β-amyrin
LjOSC1	BAE53429.1	*Lotus japonicus*	β-amyrin
MtBAS1	CAD23247.1	*Medicago truncatula*	β-amyrin
PgPNY1	BAA33461.1	*Panax ginseng*	β-amyrin
PgPNY2	BAA33722.1	*Panax ginseng*	β-amyrin
PsPSY	BAA97558.1	*Pisum sativum*	β-amyrin
SlTTS1	ADU52574.1	*Solanum lycopersicum*	β-amyrin

SAD1	CAC84558.1	*Avena species*	β-amyrin
AeBAS	ADK12003.1	*Aralia elata*	β-amyrin
TwOSC8	QBJ05694.1	*Tripterygium wilfordii*	β-amyrin
LjOSC9	BBK68801.1	*Lotus japonicus*	α-amyrin
OeOEA	BAF63702.1	*Olea europaea*	α-amyrin
BfOSC3	BBI55114.1	*Bauhinia forficata*	α-amyrin
WsOSC/LS	AGA17939.1	*Withania somnifera*	lupeol
BpBPW	BAB83087.1	*Betula platyphylla*	lupeol
BgLUS	BAF80444.1	*Bruguiera gymnorrhiza*	lupeol
GgLUS1	BAD08587.1	*Glycyrrhiza glabra*	lupeol
KdLUS	ADK35126.1	*Kalanchoe daigremontiana*	lupeol
LjOSC3	BAE53430.1	*Lotus japonicus*	lupeol
OeOEW	BAA86930.1	*Olea europaea*	lupeol
RcLUS	ABB76766.1	*Ricinus communis*	lupeol
ToTRW	BAA86932.1	*Taraxacum officinale*	lupeol
QsOSC1	QJX36710.1	*Quercus suber*	lupeol
TcOSC4	QBO24614.1	*Taraxacum coreanum*	taraxerol
AtSHS1	BAK52535.1	*Aster tataricus*	shionone
KdGLS	ADK35124.1	*Kalanchoe daigremontiana*	glutinol
TwOSC3	QHT64456.1	*Tripterygium wilfordii*	α-amyrin; β-amyrin
MdOSC1	ACM89977.1	*Malus domestica*	α-amyrin; β-amyrin
MdOSC3	ACM89979.1	*Malus domestica*	α-amyrin; β-amyrin
IaAS1	AIS39793.1	*Ilex Asprella*	α-amyrin; β-amyrin
ObAS2	AFH53506.1	*Sweet Basil*	α-amyrin; β-amyrin

CrAS	AFJ19235.1	*Catharanthus roseus*	α-amyrin; β-amyrin
IaAS2	AIS39794.1	*Ilex Asprella*	β-amyrin; α-amyrin
QsOSC2	QJX36711.1	*Quercus suber*	β-amyrin; α-amyrin
QsOSC3	QJX36712.1	*Quercus suber*	friedelin; β-amyrin
TwOSC1(2)	AWK97810.1	*Tripterygium wilfordii*	friedelin; β-amyrin; α-amyrin
TwOSC3(2)	AWK97812.1	*Tripterygium wilfordii*	friedelin; β-amyrin; α-amyrin
Bra032185	XM_009142199	*Brassica rapa*	astertarone A + 20 compounds

Table S2. Primers used in sequencing and mutation of *Monteverdia ilicifolia* friedelin synthase. Forward and reverse primers are labelled with *f* and *r*, respectively, while the mutations performed are highlighted in purple.

Gene	Nucleotide sequence	Use
Wild type	AAAAGCAGGCTTAATGTGG	Sequencing-*f*
	AATCACTCCTGAACACAGAAAG	Sequencing-*f*
	GAGACATAGATGTGCCGAAG	Sequencing-*f*
	GACAATCCTTCTGGTAACTTCG	Sequencing-*f*
	ATACCCTGACGGTTCTTGG	Sequencing-*f*
G533A	CTGGTGGTTTGGCCGCTTGGGAACCTGTTAG	Mutation-*f*
	CTAACAGGTTCCCAAGCGGCCAAACCACCAG	Mutation-*r*
Y609A	CCTGACGGTTCTTGGGCTGGTAACTGGGGTATC	Mutation-*f*
	GATACCCCAGTTACCAGCCCAAGAACCGTCAGG	Mutation-*r*
K729M	GAAATCACAGGTGTTTTTATGATGAACTGCATGTTGCAC	Mutation-*f*
	GTGCAACATGCAGTTCATCATAAAAACACCTGTGATTTC	Mutation-*r*
K729R	CAAGAAATCACAGGTGTTTTTAGAATGAACTGCATGTTGCACTATC	Mutation-*f*
	GATAGTGCAACATGCAGTTCATTCTAAAAACACCTGTGATTTCTTG	Mutation-*r*
K729W	CAACAAGAAATCACAGGTGTTTTTTGGATGAACTGCATGTTGCACTATCC	Mutation-*f*
	GGATAGTGCAACATGCAGTTCATCCAAAAAACACCTGTGATTTCTTGTTG	Mutation-*r*
K729A	GAAATCACAGGTGTTTTTGCTATGAACTGCATGTTGCAC	Mutation-*f*
	GTGCAACATGCAGTTCATAGCAAAAACACCTGTGATTTC	Mutation-*r*
K729E	GAAATCACAGGTGTTTTTGAAATGAACTGCATGTTGCAC	Mutation-*f*
	GTGCAACATGCAGTTCATTTCAAAAACACCTGTGATTTC	Mutation-*r*

Nucleotides in purple indicates the substituent nucleotides in site directed PCR reaction.

Table S3. Primers used in sequencing and mutation of *Populus davidiana* friedelin synthase. Forward and reverse primers are labelled with *f* and *r*, respectively, while the mutations performed are highlighted in purple.

Gene	Nucleotide sequence	Use
Wild type	AAAAGCAGGCTTAATGTGG	Sequencing-*f*
	TTTCCACCTGAATACAGGAAAG	Sequencing-*f*
	CCCTATGAGTCTGTCAAGTGG	Sequencing-*f*
	AGAGTCAGGTGGTAGACAATCC	Sequencing-*f*
	ATGATTCAGATGCCTGACG	Sequencing-*f*
G530A	CAAGAGGAGGATTAGCTGCTTGGGAACCAATACGTGG	Mutation-*f*
	CCACGTATTGGTTCCCAAGCAGCTAATCCTCCTCTTG	Mutation-*r*
Y606A	GCCTGACGGTTCCTGGGCTGGAAACTGGGGTGTC	Mutation-*f*
	GACACCCCAGTTTCCAGCCCAGGAACCGTCAGGC	Mutation-*r*
K7296M	GAAATCACAGGCGTCTTTATGAACAACTGTATGTTACAC	Mutation-*f*
	GTGTAACATACAGTTGTTCATAAAGACGCCTGTGATTTC	Mutation-*r*
K726R	CAAGAAATCACAGGCGTCTTTAGAAACAACTGTATGTTACACTATC	Mutation-*f*
	GATAGTGTAACATACAGTTGTTTCTAAAGACGCCTGTGATTTCTTG	Mutation-*r*
K726W	CAACAAGAAATCACAGGCGTCTTTTGGAACAACTGTATGTTACACTATCC	Mutation-*f*
	GGATAGTGTAACATACAGTTGTTCCAAAAGACGCCTGTGATTTCTTGTTG	Mutation-*r*
K726A	GAAATCACAGGCGTCTTTGCTAACAACTGTATGTTACAC	Mutation-*f*
	GTGTAACATACAGTTGTTAGCAAAGACGCCTGTGATTTC	Mutation-*r*

Nucleotides in purple indicates the substituent nucleotides in site directed PCR reaction.

Figure S1. Phylogenetic tree of OSCs.

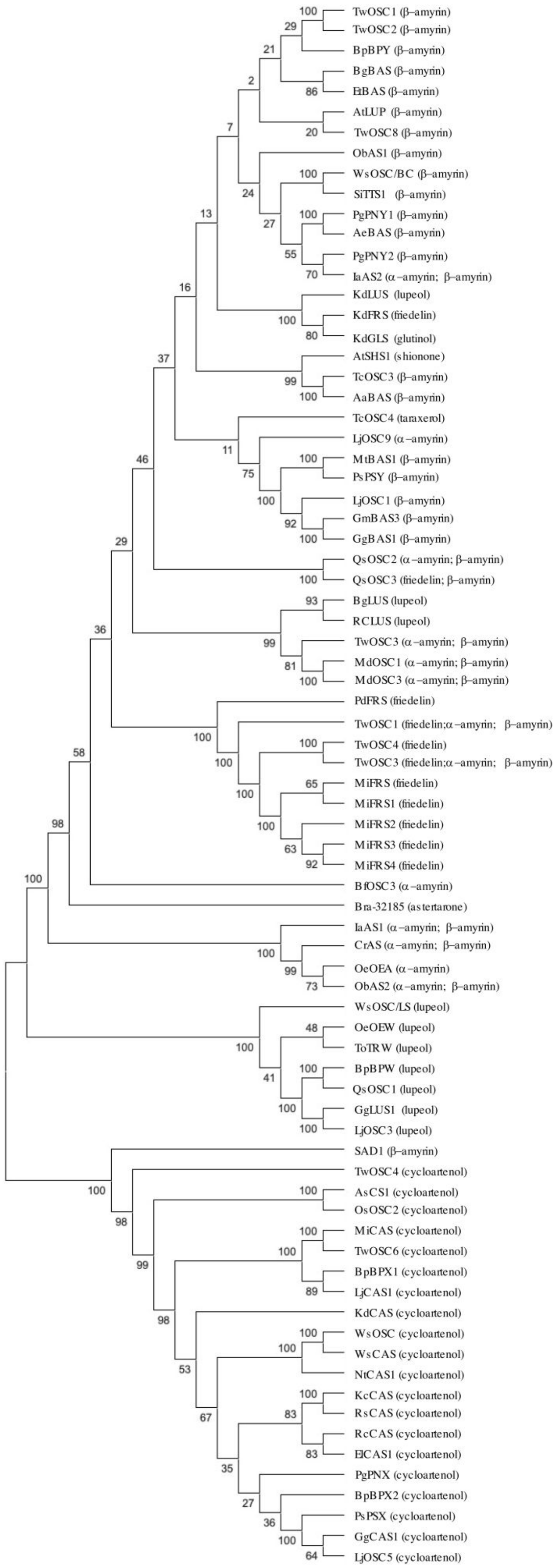

Figure S2. Logo alignment of OSCs.

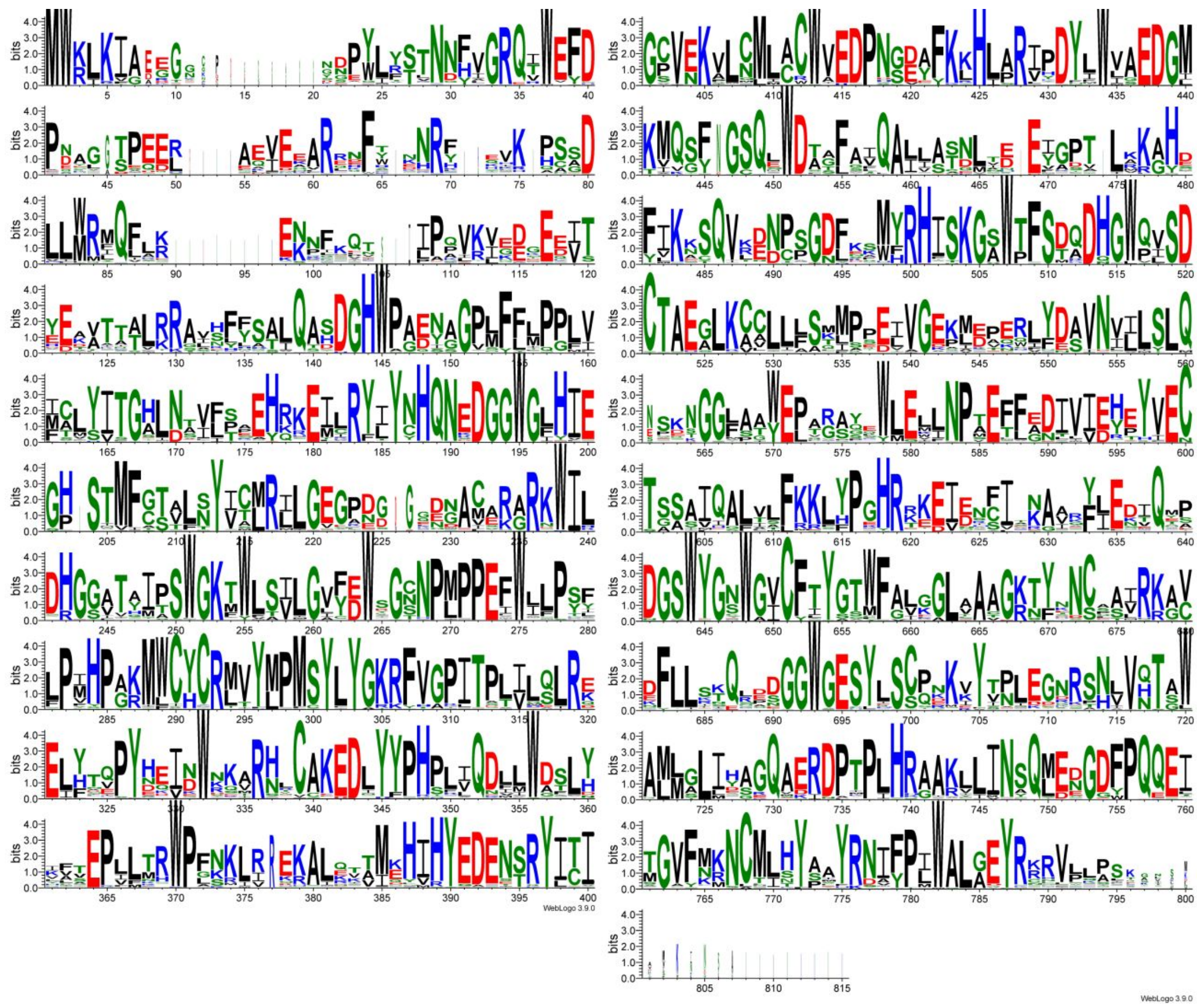

Figure S3. Multiple sequence alignment of OSCs with highlighted residues used in site-directed mutagenesis.

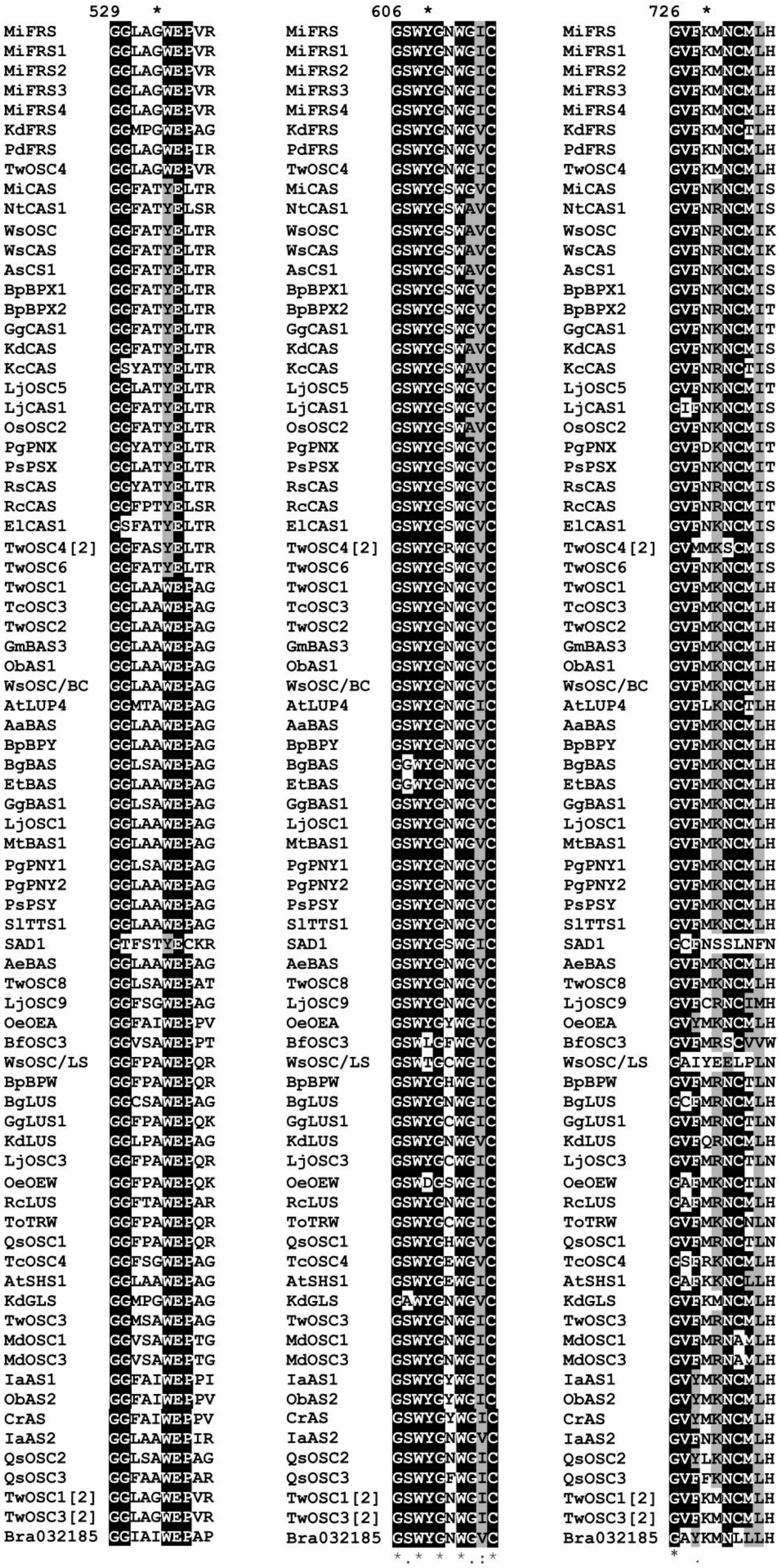

Figure S4. Chromatographic profile of *Mi*FRS wild type enzymes and mutants produced in *S. cerevisiae* using GC-MS. Friedelin is seen in T_R = 24,7 min.

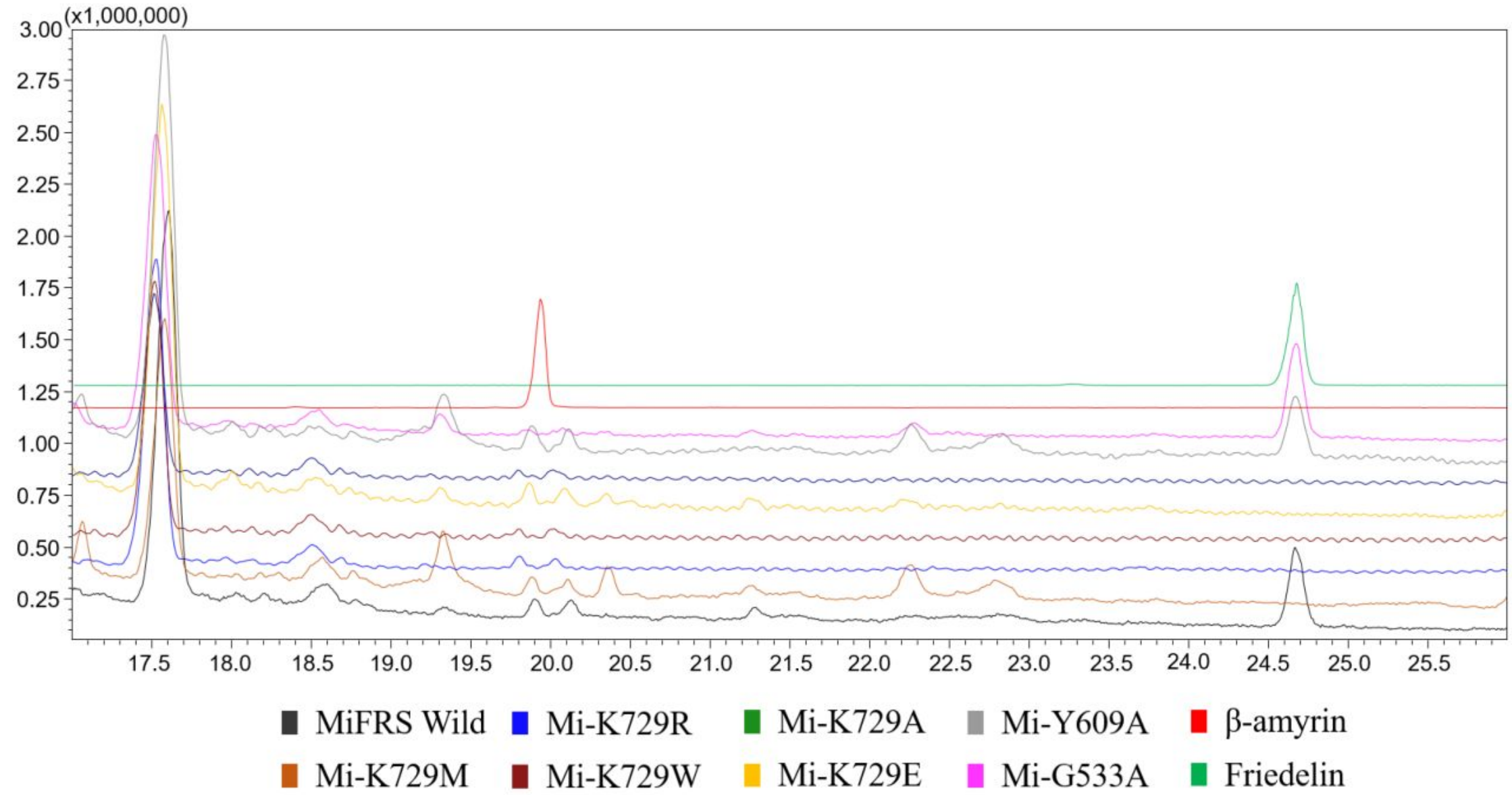

Figure S5. Chromatographic profile of *Pd*FRS wild type enzymes and mutants produced in *S. cerevisiae* using GC-MS. Friedelin is seen in T_R = 24,7 min.

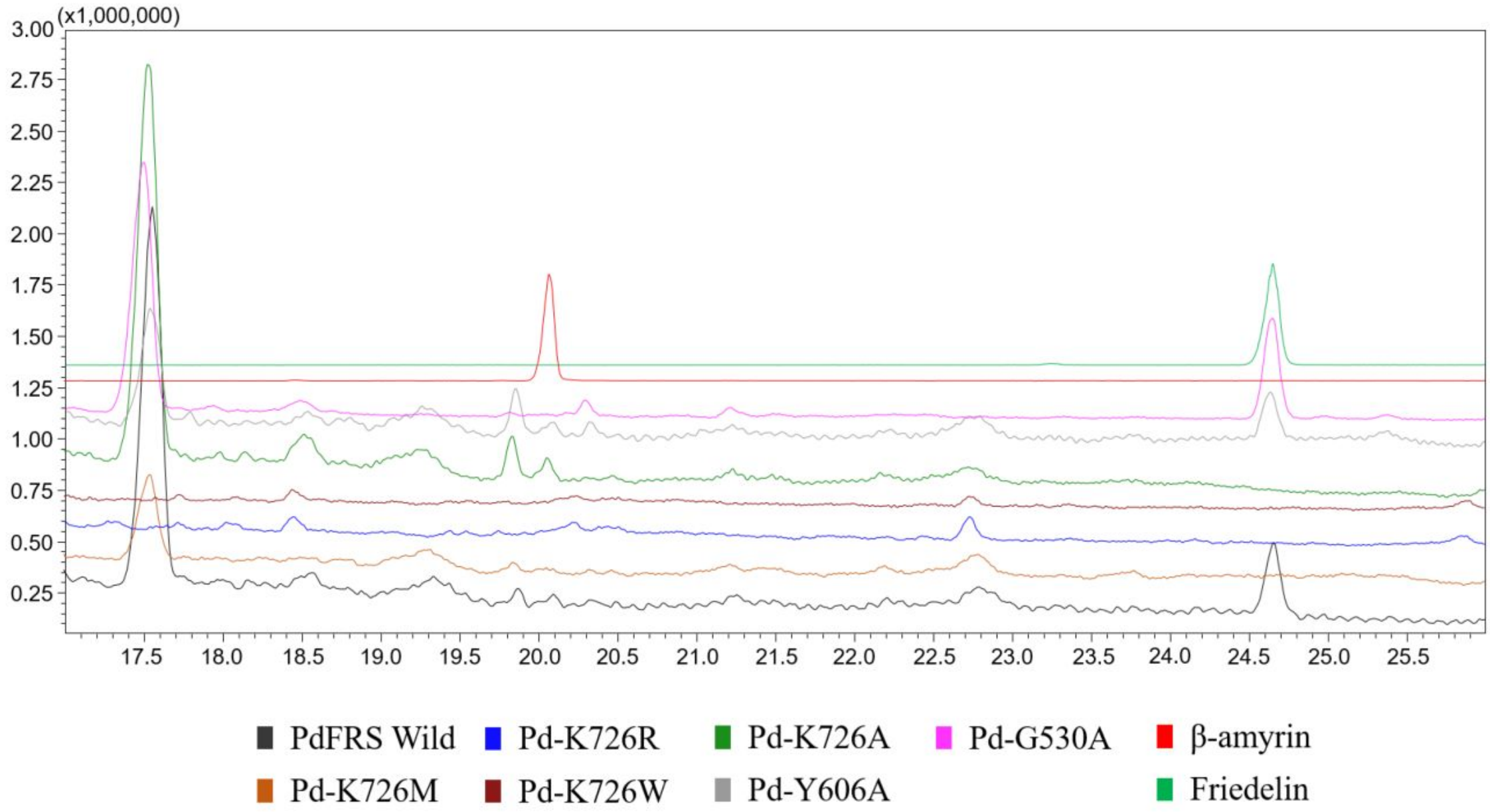

Figure S6. Root-mean-square fluctuations (a) WT system and (b) Gly533Ala mutant. For each system, three independent replicates are shown separately.

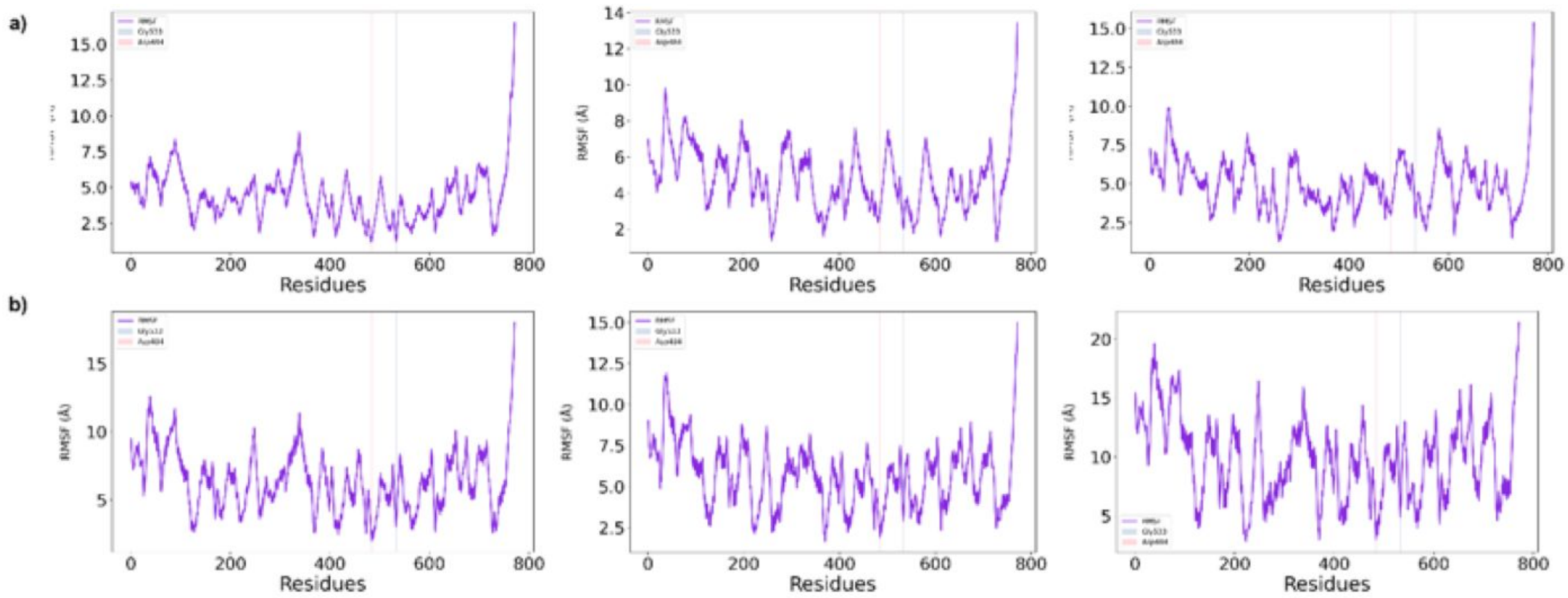

Figure S7. Root-mean-square deviation along the MD simulations of the WT system. Panels (a–c) correspond to the three independent replicates. For each replicate, the left column shows the RMSD of the entire protein–substrate complex, while the right column shows the RMSD of the substrate alone.

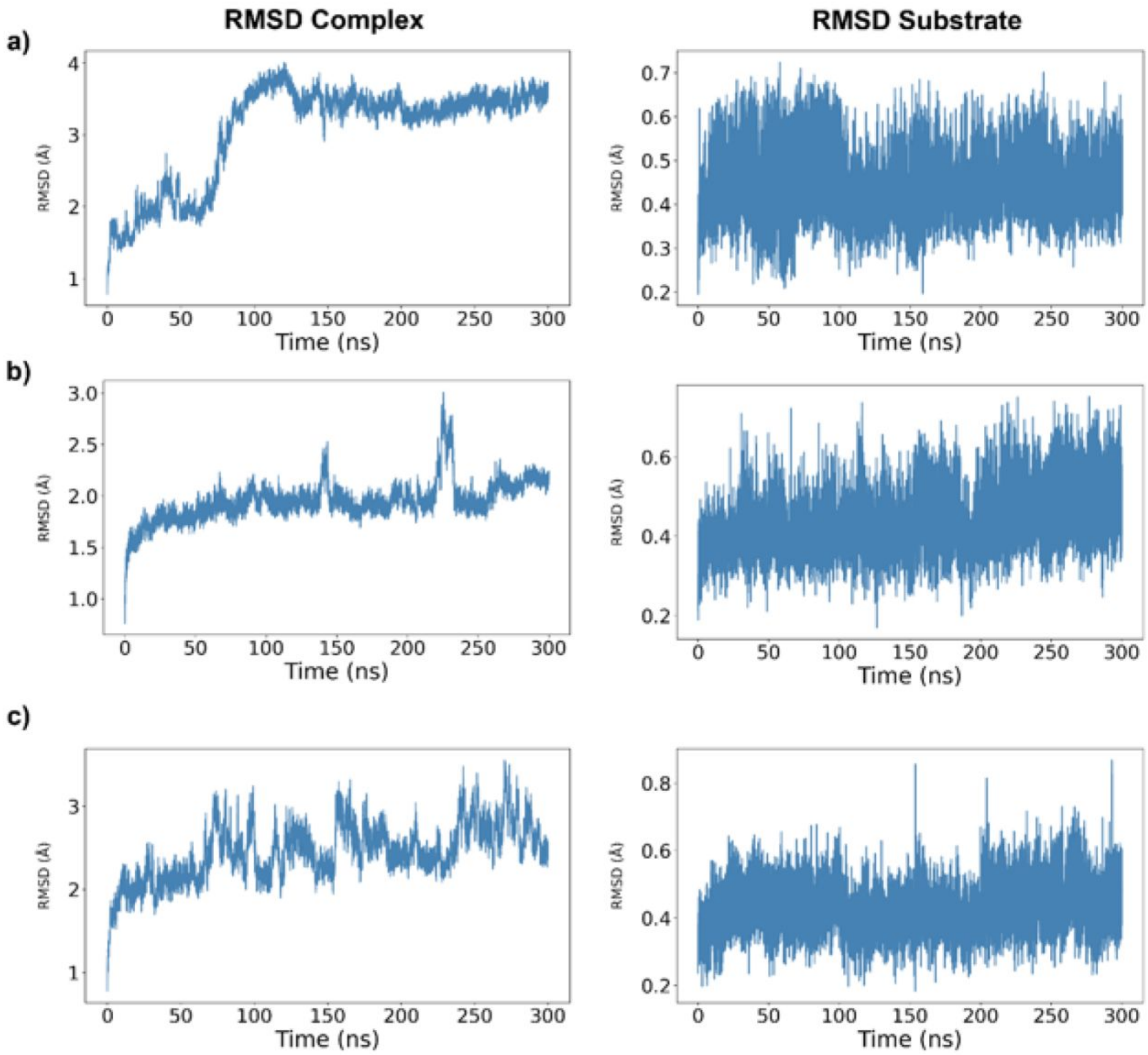

Figure S8. Root-mean-square deviation along the MD simulations of the Gly533Ala system. Panels (a–c) correspond to the three independent replicates. For each replicate, the left column shows the RMSD of the entire protein–substrate complex, while the right column shows the RMSD of the substrate alone.

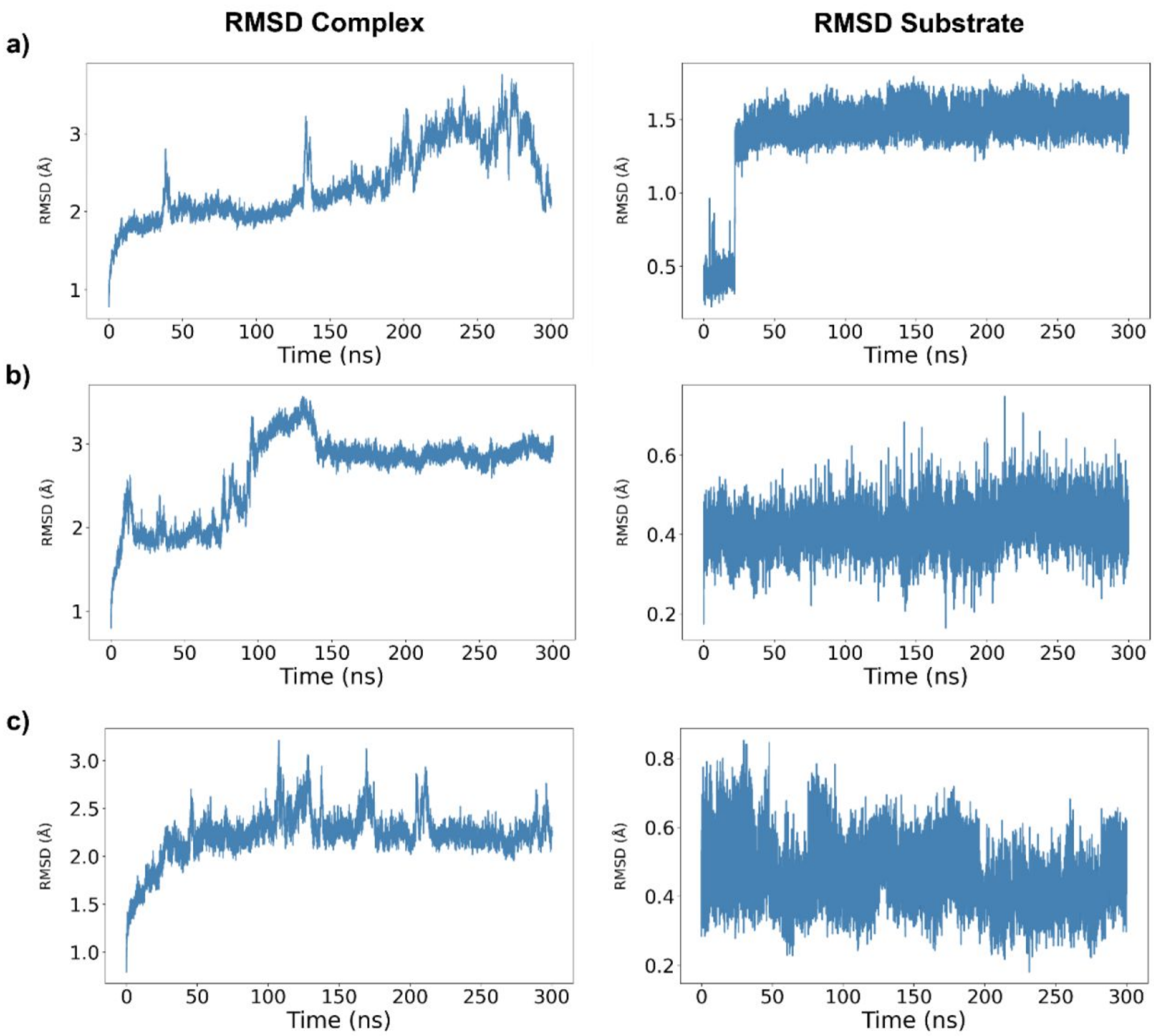

Figure S9. Distance between the hydroxyl group of the substrate and the OD1 atom of Asp484 during the MD simulations. Panel (a) corresponds to the WT system and panel (b) to the Gly533Ala mutant. For each system, the three independent replicates are shown separately.

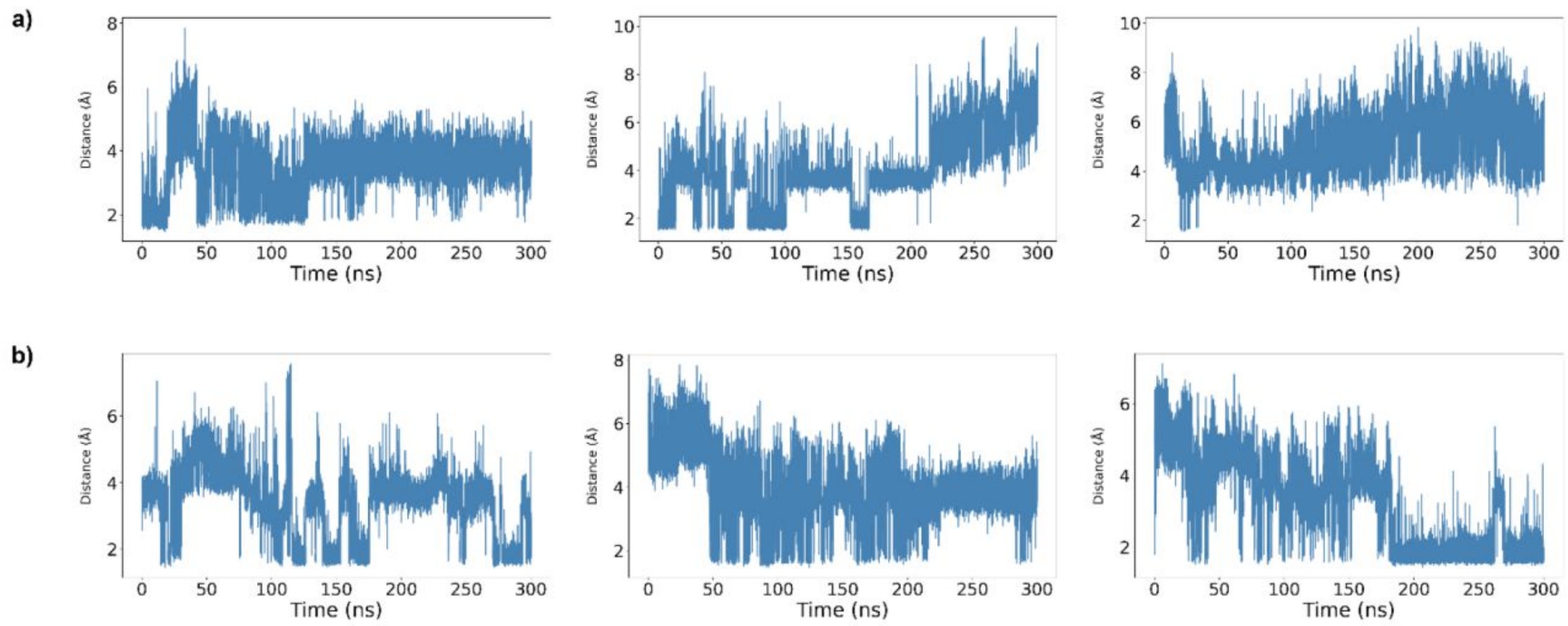

Figure S10. Distance between the hydroxyl group of the substrate and the OD2 atom of Asp484 during the MD simulations. Panel (a) corresponds to the WT system and panel (b) to the Gly533Ala mutant. For each system, the three independent replicates are shown separately.

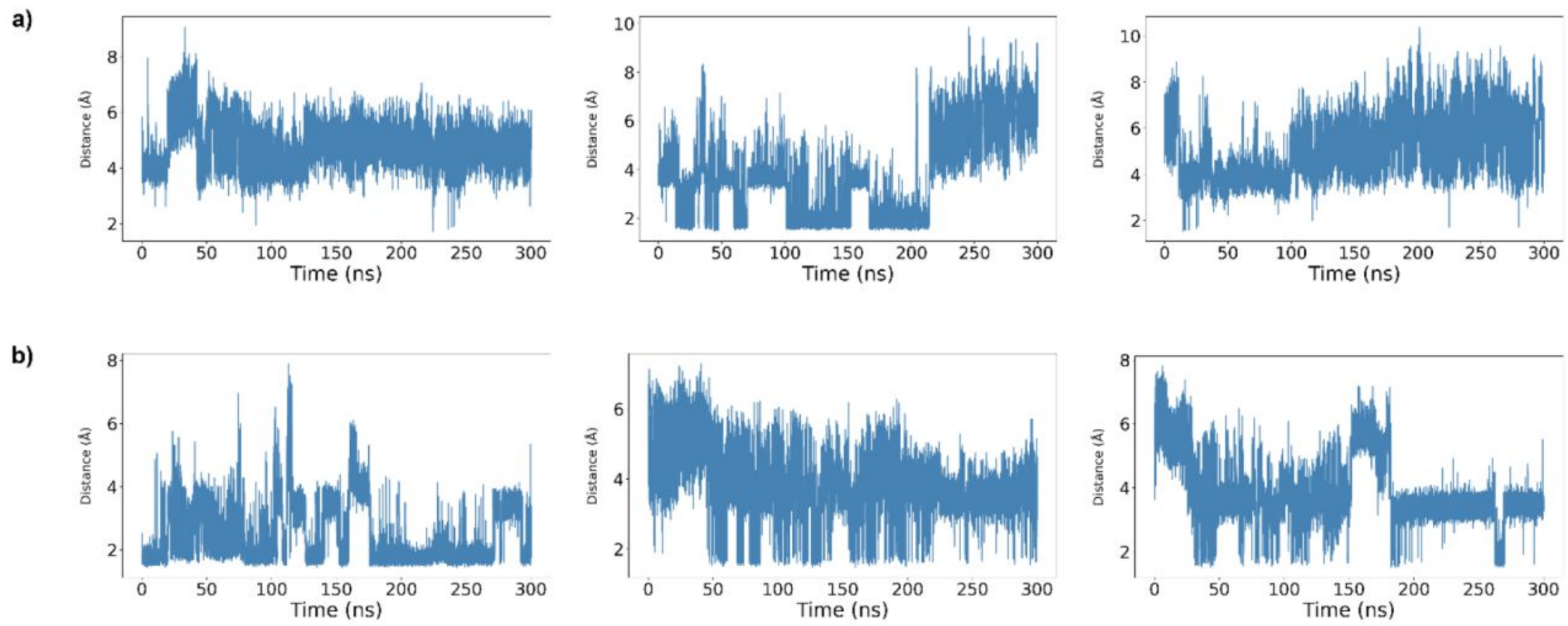

Supplement: Supplementary file 1 [file ao6c07069_si_001.pdf]
